# Moringa Extract Attenuates Inflammatory Responses and Increases Gene Expression of Casein in Bovine Mammary Epithelial Cells

**DOI:** 10.3390/ani9070391

**Published:** 2019-06-26

**Authors:** Wei Nee Cheng, Chang Hee Jeong, Han Geuk Seo, Sung Gu Han

**Affiliations:** Department of Food Science and Biotechnology of Animal Resources, Konkuk University, Seoul 05029, Korea

**Keywords:** moringa, bovine mammary epithelial cells, mastitis, mammary inflammation

## Abstract

**Simple Summary:**

Bovine mastitis, an inflammatory disease in the udder of dairy cows, is a common disease that causes low quantity and quality of bovine milk. Treatment and prevention of bovine mastitis still rely on antibiotics. However, concerns about excessive use of antibiotics have been raised due to the development of antibiotic-resistant bacteria. Therefore, natural products possessing protective effects in bovine udder have gained a lot of interests. Our objective was to investigate the possibility of *Moringa oleifera* extract (ME) in protecting bovine epithelial mammary cells. Our results demonstrated that methanol extract of *Moringa oleifera* leaves has beneficial effects in bovine mammary epithelial cells through its anti-inflammatory, antioxidant, and casein production properties. Data suggest that moringa extract could be a good feed supplement for protecting the udder of cows from inflammatory responses due to mastitis.

**Abstract:**

Bovine mastitis is a common inflammatory disease in the udder of dairy cows that causes economic loss to dairy industries. The development of alternative strategies, especially the utilization of natural products, e.g., *Moringa oleifera*, has gained a lot of interests. The objective of the current study was to investigate the protective effects of moringa extract (ME) in bovine mammary epithelial cells (MAC-T) in *in vitro* settings. Radical scavenging capacities and anti-inflammatory properties of ME were examined using lipopolysaccharide (LPS)-challenged MAC-T cells. ME showed significant radical scavenging activities. In addition, ME decreased reactive oxygen species produced by LPS in cells. ME also attenuated inflammatory cyclooxygenase-2 expression induced by LPS by down-regulating NF-κB signaling cascade. Moreover, ME ameliorated LPS-induced pro-inflammatory cytokines including tumor necrosis factor-α, interleukin-1β, and interleukin-6. Furthermore, ME up-regulated mRNA expression levels of heme oxygenase-1, NAD(P)H: quinone oxidoreductase-1, and thioredoxin reductase 1. Importantly, ME promoted differentiated MAC-T cells by increasing mRNA expression levels of α-casein S1, α-casein S2, and β-casein. In conclusion, ME has beneficial effects in bovine mammary epithelial cells through its anti-inflammatory, antioxidant, and casein production properties. Our study provides evidence that ME could be a good candidate for a feed supplement to decrease inflammatory responses due to bovine mastitis.

## 1. Introduction

Bovine mastitis, the inflammation of mammary gland and udder tissue of dairy cattle, is a common disease that causes economic losses in dairy industries [1]. Bacterial infections are the most common cause of bovine mastitis, especially *Escherichia coli*. Clinical mastitis can be diagnosed through visible symptoms such as red and swollen udder or fever in dairy cattle. Serious cases of clinical mastitis can lead to cow death. Acute inflammation responses induced by *Escherichia coli* is usually due to the endotoxin known as lipopolysaccharide (LPS) present on the outer membrane of bacteria. LPS is recognized by toll-like receptor 4 (TLR4) which then activates a series of signaling pathways. Major pathways involved in LPS challenge include mitogen-activated protein kinase, nuclear factor kappa B (NF-κB), pro-inflammatory cytokines [e.g., tumor necrosis factor-α (TNF-α), interleukin-1β (IL-1β), and Interleukin-6 (IL-6)] and other inflammatory mediators [e.g., cyclooxygenase-2 (COX-2)] [2,3]. 

Nowadays, treatment and prevention of bovine mastitis are dependent on antibiotics. Unfortunately, due to abuse of antibiotics, the development of resistant pathogens has become a global concern to veterinary and public health [4]. Therefore, the development of new control and preventive strategies is needed. For instance, the utilization of natural products has gained a lot of interests. In some developing countries, farmers have difficulties in obtaining commercial drugs. They tend to treat sick farm animals with herbal remedies known as ethnoveterinary medicine [5]. In fact, ethnoveterinary research has raised in importance in Europe as people prefer organic food to pursue a healthy lifestyle [6,7]. To search for natural substances and their active ingredients to prevent bovine mastitis, many studies have been conducted and reported, including baicalein extracted from *Scutellaria baicalensis* and *Scutellaria lateriflora* [8,9], thymol isolated from thyme, oregano, and tangerine peel [10], and curcumin from turmeric [11,12]. 

*Moringa oleifera* is a tropical plant native to India. It is commonly known as a drumstick tree. It is also known as ‘Miracle Tree’ due to its high nutrient content such as proteins, minerals, and various vitamin. All parts of moringa tree, including fruits, seeds, leaves, flowers, bark, and roots have been found to possess large amounts of beneficial nutrients [13,14]. Particularly, moringa leaf is an effective source of natural antioxidants. It contains various antioxidant compounds, including phenolic acids, flavonoids, vitamin C, tannin, saponin, phytate, oxalate, alkaloid, cardenolides, and cardiac glycosides. Thus, moringa not only provides good nutrients, but also possesses various medicinal therapeutic effects, including anti-fibrotic, anti-inflammatory, anti-microbial, anti-hyperglycemic, anti-oxidant, anti-tumor, anti-cancer, and anti-clastogenic activities [15,16]. 

The objective of this study was to examine whether moringa leave extract might have potential preventive effects on LPS-induced inflammatory responses. Due to its anti-inflammatory potential reported in previous studies, anti-inflammatory and antioxidant effects of moringa extract (ME) were investigated in the present study using bovine mammary epithelial cells, MAC-T. 

## 2. Materials and Methods 

### 2.1. Reagents

Dimethyl sulfoxide (DMSO), 3-(4,5-dimethylthiazol-2-yl)-2,5-diphenyltetrazolium bromide (MTT), and 0.4% trypan blue solution were purchased from Amresco (Solon, OH, USA). Progesterone, insulin, 2,2-diphenyl-1-picrylhydrazyl (DPPH), 2,2’-azino-bis (3-ethylbenzothiazoline-6-sulfonic acid) (ABTS), 2′,7′-dichlorofluorescein diacetate (DCF-DA), retinoic acid, hydrocortisone, and LPS from *E. coli* O111:B4 were obtained from Sigma Aldrich (St. Louis, MO, USA). Dulbecco’s modified Eagle’s medium (DMEM/high glucose and low glucose), fetal bovine serum (FBS), and penicillin/streptomycin were purchased from Welgene (Gyeongsan, Korea). Phosphate-buffered saline (PBS) and trypsin were obtained from Gibco (Grand Island, NY, USA). Primary antibodies against COX-2, NF-κB p65, proliferating cell nuclear antigen (PCNA), glyceraldehyde 3-phosphate dehydrogenase (GAPDH), and secondary antibodies against goat anti-rabbit IgG-HRP and donkey anti-goat IgG-HRP were purchased from Santa Cruz Biotechnology (Santa Cruz, CA, USA). TRIzol reagent were obtained from Life Technologies (Eugene, OR, USA). 

### 2.2. Preparation of Moringa Extract (ME)

Moringa leaf powder was purchased from Philippine Moringa & More Corporation (Rizal, Philippine). Moringa leaf powder was extracted based on a previous study [17] with minor modifications. Methanol is known to have wide solubility properties for low molecular and moderately polar substances, including antioxidant phenolic compounds [18]. Thus, methanol was chosen for extraction in this study. Moringa powder (20 g) was placed into a beaker with 200 mL of 80% (*v*/*v*) methanol. The beaker was covered with aluminum foil and stirred for 3 h at room temperature. After 3 h, the mixture was filtered with Whatman No. 1 filter paper (GE Healthcare Life Sciences, Buckinghamshire, UK). The filtered solvent was inserted into a round-bottom flask and attached to a rotary evaporator (Tokyo Rikakikai Co., Ltd., Tokyo, Japan). The solvent was evaporated under reduced pressure at 50 °C. The residue was freeze-dried and stored at -80 °C until use. Freeze-dried moringa extract (ME) was dissolved in DMSO upon usage.

### 2.3. Determination of Free Radical Scavenging Activity

ABTS and DPPH radical-scavenging activities were measured as described previously [19]. ABTS reagent (14.8 mM) was mixed with 5 mM potassium persulfate (1:1, v/v) and left in the dark for 16 h at room temperature to react. This ABTS^+^ solution was diluted with distilled water to reach absorbance of 0.700 ± 0.05 at 734 nm before use. ME at different concentrations (0-200 μg/ml) was mixed with ABTS^+^ solution in a 96-well plate and then allowed to react in the dark for 15 min at room temperature. ABTS^+^ solution added with distilled water served as a control. The absorbance wasmeasured at 734 nm. ABTS^+^ scavenging activity was calculated using the following formula:

ABTS^+^ scavenging activity (%) = [1 − (Abs_sample_/Abs_control_)] × 100%.

DPPH reagent (0.1 mM) was dissolved in ethanol and mixed with different concentrations (0–200 μg/ml) of ME in a 96-well plate and then allowed to react in the dark for 30 min at room temperature. DPPH reagent added with ethanol served as a control. After 30 min, the absorbance was read with a UV-spectrometer at a wavelength of 515 nm. DPPH scavenging activity was calculated using the following formula:

DPPH scavenging activity (%) = [1 − (Abs_sample_/Abs_control_)] × 100%.

### 2.4. Cell Culture and Treatments

The MAC-T cell line was obtained from Prof. Hong Gu Lee (Konkuk University, Seoul, Korea). MAC-T cells were cultured in high glucose DMEM containing 10% FBS, penicillin/streptomycin, 5 μg/ml insulin, and 1 μg/ml progesterone in a CO_2_ incubator at 37 °C. Cells were first grown to 90–100% confluency and then pre-treated with ME at concentrations of 50 and 200 μg/ml for 12 h. These ME-treated cells were then treated with LPS (1 μg/ml) for 6 or 12 h in order to investigate the anti-inflammatory effect of ME. LPS concentration was based on previous studies using bovine mammary epithelial cells [3,20]. 

Differentiation of MAC-T was conducted according to previous reports [21,22] with some modifications. Briefly, MAC-T cells were grown to 90% confluency in medium as described earlier. After cells were starved with serum-free DMEM for 16 h, cells were cultured in low glucose DMEM containing 5% FBS, penicillin/streptomycin, 5 μg/ml insulin, 1 μg/ml of hydrocortisone, 5 μg/ml of prolactin, and 1 μM of retinoic acid in a CO_2_ incubator at 37 °C. Medium was changed daily for eight days. To determine casein mRNA expression, 200 μg/ml of ME was added to cells during the last medium change. After 12 h, LPS was used for treatment for another 12 h before cells were harvested. 

### 2.5. Cytotoxicity Test

Cytotoxicity of ME was determined by MTT assay. MAC-T cells were seeded in a 96-well plate and treated with ME (0-400 μg/ml) for 24 h. The optical density of the 96-well plate was then measured with a UV-spectrophotometer (Biotek Instrument, USA) at 570 nm. Trypan blue exclusion assay was conducted for further confirmation. MAC-T cells were grown in 6-well plates and treated with ME (0-400 μg/ml) followed by incubation for 24 h. Cells were detached with trypsin and dyed with trypan blue. Viable cells were counted using a haemocytometer.

### 2.6. Preparation of Cell Lysate, SDS-PAGE, and Western Blot Analysis

For total protein collection, cells were lysed with RIPA-buffer containing 50 mM Tris (pH 8.0), 150 mM NaCl, 1% Triton X-100, 0.5% sodium deoxycholate, 0.1% SDS, and a protease inhibitor mixture (2 μg/ml aprotinin, 10 μg/ml leupeptin, 1 mM PMSF, 5 mM EDTA, 1 mM EGTA, 10 mM NaF, and 1 mM Na_3_VO_4_). Lysed cells in 6-well plates were collected with cell scrappers and centrifuged at 23,500 × g for 20 min at 4 °C. Supernatants were collected and protein concentrations were analyzed using Pierce BCA protein assay kit (Sigma-Aldrich, St. Louis, MO, USA). Cell lysates were stored at −80 °C until further use. For Western blot analysis, protein samples were separated using SDS-PAGE and transferred onto nitrocellulose membranes. These membranes were then blocked with 3% skim milk buffer for 1 h 30 min at room temperature and washed with Tris-buffered saline (TBS). Membranes were incubated with primary antibody at 4 °C overnight. After washing with TBS, membranes were incubated with appropriate secondary antibodies conjugated with horseradish peroxidase for 2 h at room temperature and visualized using ECL detection reagents (Waltham, MA, Thermo Scientific, USA).

### 2.7. Nuclear Fractionation

Nuclear translocation detection of NF-κB p65 was performed as described previously [23]. Briefly, cells were grown in cell culture dishes and treated with ME (50 and 200 μg/mL) for 12 h followed by treatment with LPS (1 μg/ml) for 6 h. Cells were lysed with a hypotonic buffer solution containing 20 mM Tris (pH 7.4), 10 mM NaCl, 3 mM MgCl_2_, and a protease inhibitor mixture. After addition of 10% Triton-X 100, cell lysates were centrifuged at 650 × g for 10 min at 4 °C and supernatants were collected as cytosolic fractions. Remaining pellets were resuspended in cell extraction buffer [100 mM Tris (pH 7.4), 1% Triton X-100, 10% glycerol, and 0.1% SDS] containing protease inhibitor mixture. Homogenates were then centrifuged at 14,000 × g for 20 min at 4 °C and supernatants were collected as nuclear fractions. These nuclear fractions were then analyzed by SDS-PAGE followed by Western blot using antibody against NF-κB p65. PCNA, a nucleus-specific housekeeping protein, was used as a loading control.

### 2.8. Real-Time PCR Analysis

Cells were grown in 6-well plates and total RNAs were extracted using TRIzol reagent (Life Technologies) according to the manufacturer’s protocol. Reverse transcription was carried out using TOPscript RT DryMIX kit (Enzynomics, Daejeon, Korea). To determine mRNA expression levels, real-time PCR was performed using Roche LightCycler^®^ 96 System (Basel, Switzerland) and 2× Real-Time PCR mix (SolGent, Daejeon, Korea). PCR conditions were as follows: initial denaturation at 95 °C for 15 min, followed by 45 cycles of amplification at 95 °C for 20 sec and 60 °C for 40 sec. Final extension at 60 °C for 60 sec and a hold at 4 °C were then performed. Data analysis was performed using the relative quantification method (∆∆Cq), in which relative mRNA expression of target mRNAs [i.e., TNF-α, IL-6, IL-1β, heme oxygenase-1 (HO-1), NAD(P)H: quinone oxidoreductase-1 (NQO-1), and thioredoxin reductase 1 (TXNRD1) and casein isoforms] was compared to that of a constitutively expressed gene (i.e., GAPDH). Primer sequences used in this study are shown in Table 1.

### 2.9. Assessment of Reactive Oxygen Species (ROS)

Intracellular ROS level was measured as described previously with some modification [19]. Briefly, cells were grown in 6-well plates until 90% confluency. Cells were treated with ME for 12 h followed by treatment with LPS for 4 h. Cells were then incubated with DCF-DA (10 μM) for 30 min and washed with PBS. The 6-well plates with cells were visualized with an Eclipse Ti2-U fluorescent microscope (Nikon Co. Ltd., Tokyo, Japan) at 200x magnification. The fluorescent area was quantified by Image J software.

### 2.10. Statistical Analysis

Data are expressed as mean ± standard error of the mean (SEM). Statistical significance was determined with Student’s t-test using SPSS-PASW statistics software ver. 18.0 for Windows (SPSS, USA). A probability value of *p* < 0.05 was considered statistically significant.

## 3. Results

### 3.1. Free Radical Scavenging Activities of ME

**Figure 1:**
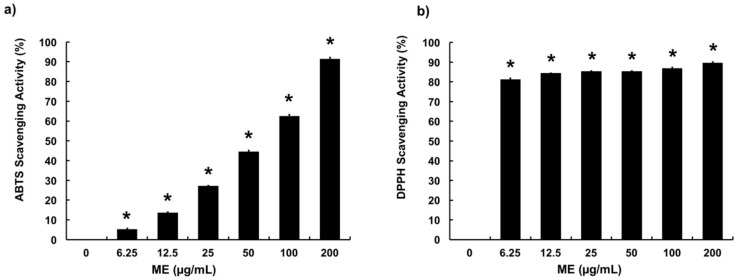
Radical scavenging activities of moringa extract (ME). (**a**) ABTS and (**b**) DPPH radical scavenging assays were performed to determine antioxidant activity of ME at concentrations of 0–200 µg/mL. Values represent means ± SEM (*n* = 5). *, significant difference vs. control (*p* < 0.05).

ME was examined for its radical scavenging capacities using ABTS and DPPH radical scavenging assays. ME showed significant radical scavenging activities in both assays. In ABTS assay, results showed that ME at concentrations of 6.25–200 μg/mL scavenged 5–90% of ABTS^+^, in a dose-dependent manner (Figure 1a). Moreover, ME showed high radical scavenging capacity in DPPH assay (Figure 1b). Particularly, ME at a concentration as low as 6.25 μg/mL showed 81% of DPPH scavenging activity, while 12.5, 25, 50, 100, and 200 μg/mL showed approximately 90% of DPPH scavenging capacity. These results indicate that ME possesses significant antioxidant properties.

### 3.2. Cytotoxicity of ME in MAC-T Cells

**Figure 2:**
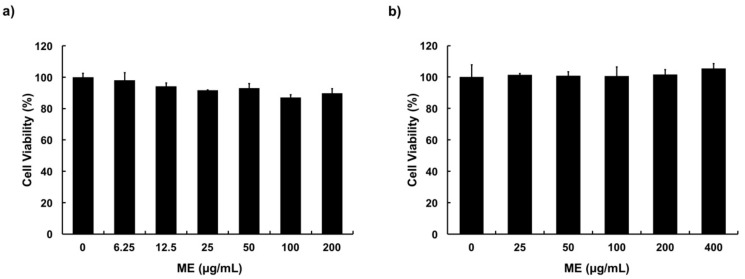
Cytotoxicity of moringa extract (ME) in MAC-T cells. (**a**) MTT assay and (**b**) trypan blue exclusion assay were used to determine the viability of MAC-T cells. Cells were treated with various concentrations (0–400 µg/mL) of ME for 24 h. Values represent means ± SEM (*n* = 5).

Cytotoxicity of ME toward MAC-T cells was determined through MTT assay and trypan blue dye exclusion assay. Results showed that ME had no cytotoxic effect on MAC-T cells at concentration up to 400 μg/ml, compared with the control after 24 h of treatment (Figure 2a,b). 

### 3.3. Anti-Inflammatory Effects of ME in MAC-T Cells

**Figure 3:**
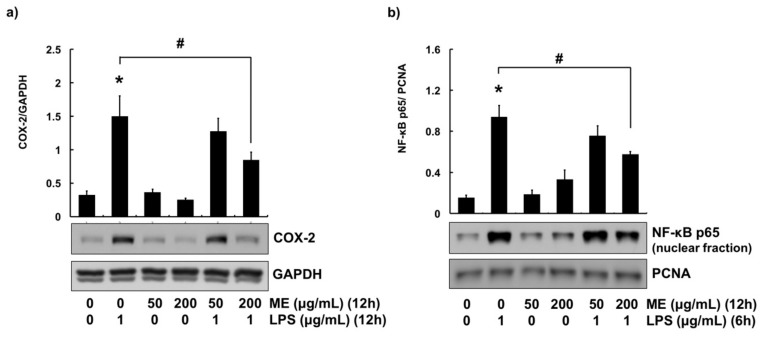
COX-2 expression and NF-κB activation in cells treated with moringa extract (ME). (**a**) Cells were pre-treated with ME (50 and 200 µg/mL) for 12 h followed by LPS treatment (1 µg/mL) for 12 h. Western blot analysis was used to measure COX-2 expression levels in whole cell lysates. GAPDH was used as a loading control. (**b**) Cells were pre-treated with ME (50 and 200 µg/mL) for 12 h followed by LPS treatment (1 µg/mL) for 6 h. Nuclear translocation of NF-κB p65 was determined using nuclear fraction and Western blot analysis. PCNA was used as a loading control. Western blots shown are representative images of three independent experiments. Values represent means ± SEM (*n* = 3). *, significant difference vs. control (*p* < 0.05). #, significant difference vs. LPS only (*p* < 0.05).

COX-2 is an enzyme associated with many inflammatory diseases. It is often used as an indicator for inflammation in *in vitro* studies using bovine mammary epithelial cells [3,20,24,25]. To determine the anti-inflammatory effects of ME, cells were treated with 1 μg/mL of LPS. The protein expression level of COX-2 was then examined by Western blotting. Protein expression of COX-2 was induced in cells treated with LPS. However, pre-treatment of cells with 200 μg/mL of ME significantly attenuated LPS-induced expression of COX-2 (Figure 3a). 

Since NF-κB signaling is an important pathway in regulating the expression of COX-2, its activation was determined using nuclear fractionation technique [3,17]. To explore whether NF-κB pathway was modulated by ME, cells were pre-treated with ME (50 and 200 μg/mL, 12 h), followed by LPS challenge for 6 h. Similar with COX-2 expression, LPS increased translocation of NF-κB p65 subunit into the nucleus. In contrast, the nuclear translocation of NF-κB p65 subunit was decreased in cells treated with 200 μg/mL of ME (Figure 3b). 

**Figure 4:**
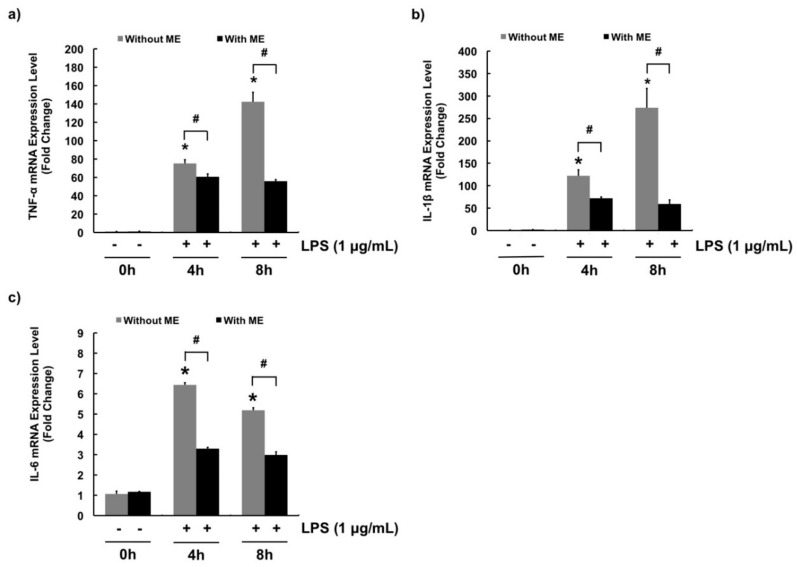
mRNA expression levels of pro-inflammatory cytokines in cells treated with moringa extract (ME). Gene expression levels of pro-inflammatory cytokines (**a**) TNF-α, (**b**) IL-1β, and (**c**) IL-6 were determined. Cells were pre-treated with or without ME (200 µg/mL) for 12 h followed by LPS treatment (1 µg/mL) for 0, 4, and 8 h. mRNA expression levels were measured using RT-PCR. mRNA expression levels were calculated relative to GAPDH expression. Values represent means ± SEM (*n* = 3). *, significant difference vs. control (no ME, no LPS) (*p* < 0.05). #, significant difference between the two treatment groups (*p* < 0.05).

Quantitative RT-PCR was performed to determine mRNA expression levels of pro-inflammatory cytokines such as TNF-α, IL-1β, and IL-6. LPS significantly increased mRNA expression levels of these pro-inflammatory cytokines (Figure 4a–c). In Figure 4a, the mRNA expression of TNF-α significantly increased in cells treated with LPS, while pre-treatment of cells with ME decreased the expression. Likewise, ME significantly decreased LPS-induced mRNA expression of IL-1β and IL-6 (Figure 4b,c).

### 3.4. Antioxidant Effects of ME in MAC-T Cells

**Figure 5:**
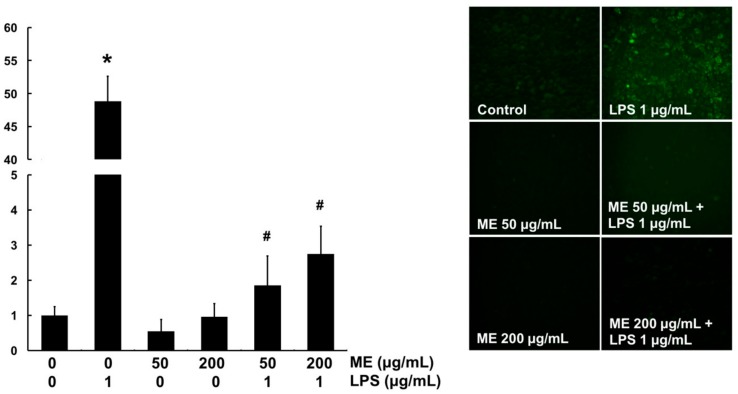
Intracellular ROS scavenging activity of moringa extract (ME). Cells were treated with ME for 12 h and then stimulated with LPS for 4 h. Cells were then stained with DCF-DA to detect ROS production. The intensity of green fluorescence was assessed using a fluorescence microscope at 200× magnification. Images shown are representatives of three independent experiments. Values represent means ± SEM (*n* = 3). *, significant difference vs. control (*p* < 0.05). #, significant difference vs. LPS only (*p* < 0.05).

**Figure 6:**
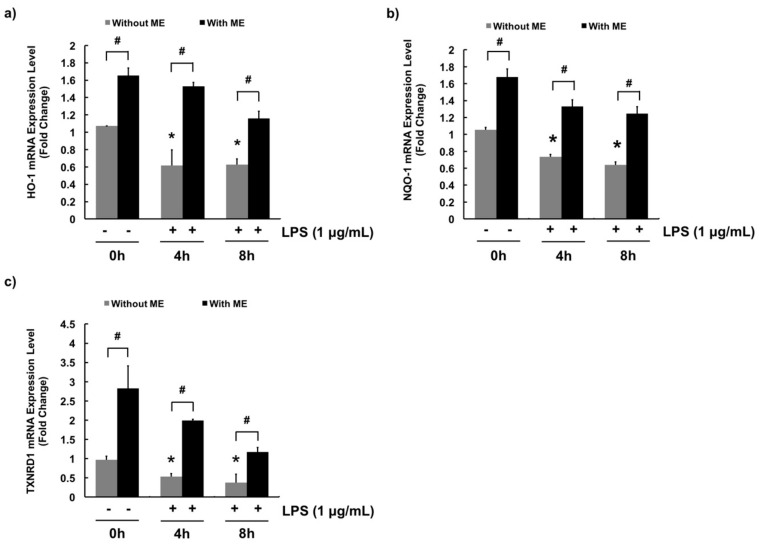
Moringa extract (ME) increases mRNA expression levels of anti-oxidant enzymes in MAC-T cells. mRNA expression levels of (**a**) HO-1, (**b**) NQO-1, and (**c**) TXNRD1 were determined. Cells were pre-treated with or without ME (200 µg/mL) for 12 h followed by LPS treatment (1 µg/mL) for 0, 4, and 8 h. mRNA expression levels were measured using RT-PCR. mRNA expression levels were calculated relative to GAPDH expression. Values represent means ± SEM (*n* = 3). *, significant difference vs. control (No ME, no LPS) (*p* < 0.05). #, significant difference between the two treatment groups (*p* < 0.05).

*In vitro* ROS scavenging activity of ME was determined using DCF-DA fluorescent dye in MAC-T cells. DCF-DA will be deacetylated to a non-fluorescent compound once diffused into the cells. However, it will later be oxidized by intracellular ROS and form a highly fluorescent compound which generates a green fluorescence. Therefore, the green area represents the production of ROS. The level of ROS was significantly increased by LPS compared with the control (Figure 5). However, cells pre-treated with ME at both 50 and 200 μg/mL showed markedly decreased ROS level in LPS-challenged cells (Figure 5). These results indicate that ME has an antioxidant effect against LPS-induced ROS production in MAC-T cells. For further confirmation, mRNA expression levels of antioxidant genes such as HO-1, NQO-1, and TXNRD1 were examined using quantitative RT-PCR. As expected, mRNA expression levels of these antioxidant genes were decreased upon LPS stimulation whereas they were significantly increased in cells pre-treated with ME (Figure 6). 

### 3.5. Effects of ME on Casein Production in Differentiated MAC-T Cells 

**Figure 7:**
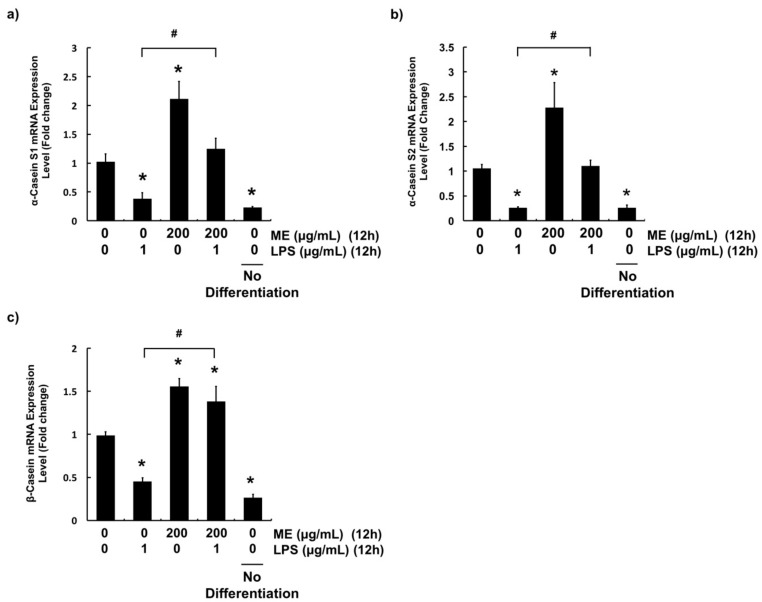
Gene expression levels of casein isoforms in differentiated MAC-T cells. mRNA expression levels of (**a**) α-Casein S1 (**b**) α-Casein S2, and (**c**) β-Casein were determined. Cells were differentiated for 8 d and treated with or without ME (200 µg/mL) for 12 h followed by LPS treatment (1 µg/mL) for 12 h. mRNA expression levels were measured using RT-PCR. mRNA expression levels were calculated relative to GAPDH expression. Values represent means ± SEM (*n* = 3). *, significant difference vs. control (No ME, no LPS) (*p* < 0.05). #, significant difference between the two treatment groups (*p* < 0.05). ME = moringa extract.

To evaluate whether ME could affect the synthesis of milk components, mRNA expression level of casein in MAC-T cells was examined. After cells were grown in differentiation media for eight days, they were pre-treated with 200 μg/mL of ME for 12 h followed by LPS challenge for 12 h. Gene expression levels of α-casein S1, α-casein S2, and β-casein were evaluated with quantitative RT-PCR. After stimulation with LPS for 12 h, mRNA expression levels of three casein isoforms (α-casein S1, α-casein S2, and β-casein) were significantly decreased (Figure 7). In contrast, pre-treatment of cells with ME recovered their gene expression levels to the control levels (α-casein S1, α-casein S2) or more (β-casein). Importantly, treatment with ME induced significant casein gene expression in differentiated cells compared to control (Figure 7).

## 4. Discussion

Bovine mastitis is an intra-mammary infection of mammary glands and udder tissue of dairy cattle that causes economic losses in dairy industries [1]. Bovine mastitis is often associated with bacterial infections. It is usually treated or prevented by antibiotics. However, antibiotic is no longer the most desirable treatment option [4]. Moreover, antibiotic residue in milk can get into human food-chain through milk consumption, bringing negative effects on human health. Therefore, new control and preventive strategies using natural products have been gaining attention in dairy industries. 

*M. oleifera* has been used to combat malnutrition, especially in infants and breastfeeding mothers in many developing countries [26,27]. In fact, feeding moringa leaves to dairy cattle not only increases milk yield, but also improves their health [28,29,30]. However, the effect of moringa on bovine mastitis has been rarely reported. Therefore, in this study, ME was examined for its anti-inflammatory, antioxidant, and casein production properties in bovine mammary epithelial cells. We hypothesized that ME had an anti-inflammatory effect due to its rich anti-antioxidant compounds. In addition, healthy mammary cells can produce more milk components such as casein. Of all detected polyphenols in moringa, the predominant groups have been reported to be kaempferol and quercetin derivatives [18,31,32,33,34]. 

In the current study, methanol was used to extract moringa leaves. Methanol is a more desirable solvent to extract polyphenol compounds from moringa leaves than other solvents (e.g., ethyl acetate, dichloromethane, and *n*-hexane) as described previously [35]. ME showed a significant radical scavenging property in both ABTS and DPPH radical scavenging assays. In DPPH radical scavenging assay, ME at low concentration showed strong radical scavenging activities. This might be due to the different solubility of ABTS^+^ and DPPH. As a methanolic extract, ME was more readily dissolved to react with free radicals in the organic phase (DPPH) than that in the aqueous phase (ABTS) [36,37].

MAC-T cells are clonal cell line of bovine mammary epithelial cells established by transfecting cells with simian virus-40 large T-antigen, to give the cells immortality and able to be cultured more than 350 passages [38]. MAC-T cells are frequently used to study bovine mammary inflammation and mastitis as an *in vitro* model [3,20,24,39]. This is because MAC-T cells and primary bovine mammary epithelial cells show similar biological responses [39]. Thus, MAC-T cells were employed to elucidate cell responses in this study. ME was examined for its cytotoxicity using MTT and trypan blue exclusion assays to obtain suitable treatment concentrations. ME showed no cytotoxic effects on MAC-T cells at a concentration up to 400 μg/mL. Therefore, concentrations of 50 and 200 μg/mL of ME were chosen for subsequent experiments, representing low and high concentration treatments, respectively. 

To determine the anti-inflammatory effect of ME, the expression level of COX-2 was evaluated using Western blot analysis. COX-2 is an inducible enzyme activated upon extracellular and intracellular physiological stimuli such as LPS and TNF-α [40]. In particular, overexpression of COX-2 is often used as an indicator of inflammation in *in vitro* studies [3,20,24,25]. COX-2 expression was highly increased by LPS challenge (1 μg/ml). However, such an increase was attenuated by 200 μg/mL of ME. Previously, down-regulation of COX-2 by moringa extract was observed in other cell lines such as RAW 264.7 [41,42] and MCF-7 cells [43], in agreement with our data. Furthermore, the expression of COX-2 was regulated via NF-κB pathway in numerous studies using bovine mammary cells [3,20,24,44]. 

NF-κB is a heterodimer composed of p65 and p50 subunits [2,44]. Under normal circumstances, NF-κB heterodimer stays in the cytoplasm in an inactive form binding with an inhibitor of kappa-B alpha (IκBα). Upon stimulation, IκBα is degraded and releases NF-κB, then NF-κB is translocated to the nucleus [2]. Therefore, the activation of NF-κB (nuclear translocation of p65subunit) was evaluated in the present study. LPS-induced activation of NF-κB p65 subunit was blocked by pre-treatment with ME. Our results indicate that ME can down-regulate the expression of COX-2 through suppression of NF-κB pathway.

In fact, activation of NF-κB induced not only COX-2 expression, but also the transcription of pro-inflammatory genes such as TNF-α, IL-1β, and IL-6. These pro-inflammatory cytokines play an important role in initiating inflammatory responses and recruiting leukocytes (e.g., neutrophils and macrophages) to target sites [45]. Indeed, mRNA expression levels of pro-inflammatory genes also represent inflammatory responses. As reported in multiple studies about bovine mastitis, plant constituents such as baicalein [8], curcumin [12], and magnolol [46] can down-regulate pro-inflammatory cytokines by blocking activation of NF-κB. Thus, in this study, ME was examined for its ability to decrease pro-inflammatory cytokines. As expected, TNF-α, IL-1β, and IL-6 gene levels were induced in cells upon stimulation with LPS. However, their levels were decreased by pre-treatment of cells with ME. Our data demonstrate that treatment with ME can protect bovine mammary epithelial cells against inflammation.

Oxidative stress is associated with bovine mastitis. For example, high-producing dairy cattle tend to accumulate ROS due to intensive cell metabolism [24,47]. Continuously generation of ROS can lead to tissue damage and acute inflammatory responses that can result in bovine mastitis. Cellular antioxidant mechanisms such as expression of antioxidant enzymes can minimize oxidative stress. Emerging evidence suggests that the anti-inflammatory effect of ME might be attributed to its antioxidant properties [48,49,50]. To further explore this association, the antioxidant effects of ME were examined. Our ROS detection assay (DCF-DA assay) showed that ME was capable of scavenging intracellular ROS produced by LPS. Such antioxidant effect of ME in cells might be due to its ROS scavenging effects observed in DPPH and ABTS^+^ scavenging assays. In addition, whether ME had regulatory effects on the expression of HO-1, NQO-1, and TXNRD1 was examined. These antioxidant proteins are phase II detoxifying enzymes that provide intracellular defensive mechanism [51,52]. Our data showed that mRNA expression levels of these three antioxidant genes were increased in cells treated with ME, indicating that they are involved in the intracellular antioxidant mechanism of ME. These findings were in agreement with other studies showing that moringa extract could inhibit the production of pro-inflammatory cytokines and induce the production of antioxidant enzymes in rats [49,53]. In fact, these antioxidant effects were attributed to the rich content of flavonoids (e.g., myricetin, quercetin, and kaempferol) and phenolic acids (e.g., gallic acid and chlorogenic acid) in ME. They are well-known antioxidants that can help neutralize free radicals, quench singlet or triplet oxygen, or decompose peroxides [54,55].

Lactation is the most important function of bovine mammary epithelial cells. Lactation involves a hormone called prolactin that is secreted by lactotroph cells of the anterior pituitary gland [56,57]. In particular, prolactin plays an important role in mammary gland development during pregnancy and lactogenesis [58,59]. It has been reported that MAC-T cells can secret more β-casein when they are induced by prolactin, together with retinoic acid, hydrocortisone, and insulin [21,22,60]. Complete mammary epithelial cell differentiation is defined by sequential activation of genes coding for milk proteins [61]. However, bovine mastitis causes cellular damage that can lead to disruption in lactation. Since casein synthesis serves as an indicator of lactation, mRNA expression levels of casein genes (α-casein S1, α-casein S2, and β-casein) were evaluated with quantitative RT-PCR [62,63]. Results demonstrated that ME could protect mammary epithelial cells against LPS-induced down-regulation of casein genes. This might be linked to the anti-inflammatory and antioxidant effects of ME in cells. Differentiated MAC-T cells treated with ME showed increased expression levels of three casein isoforms. These data suggest that ME can stimulate the production of milk proteins in healthy mammary alveolar epithelial cells. A previous study has reported that consumption of moringa leaf can increase milk production in white female Wistar rats [64]. Indeed, in several studies, dairy cattle fed with ensiled moringa showed higher milk yield and lower somatic cell counts [28,29,30]. Our data elucidated the underlying mechanism about the beneficial role of moringa at cellular and molecular levels. Based on these results, phytosterols present in ME including stigmasterol, sitosterol, and kaempesterol might be contributors to the expression of casein genes. Phytosterols can act as precursors of hormone such as prolactin and estrogen that can stimulate mammary cells to produce milk components [61]. Taken together, our data provide evidence that moringa has potential to promote udder health and production of milk components in dairy cattle.

## 5. Conclusions

**Figure 8:**
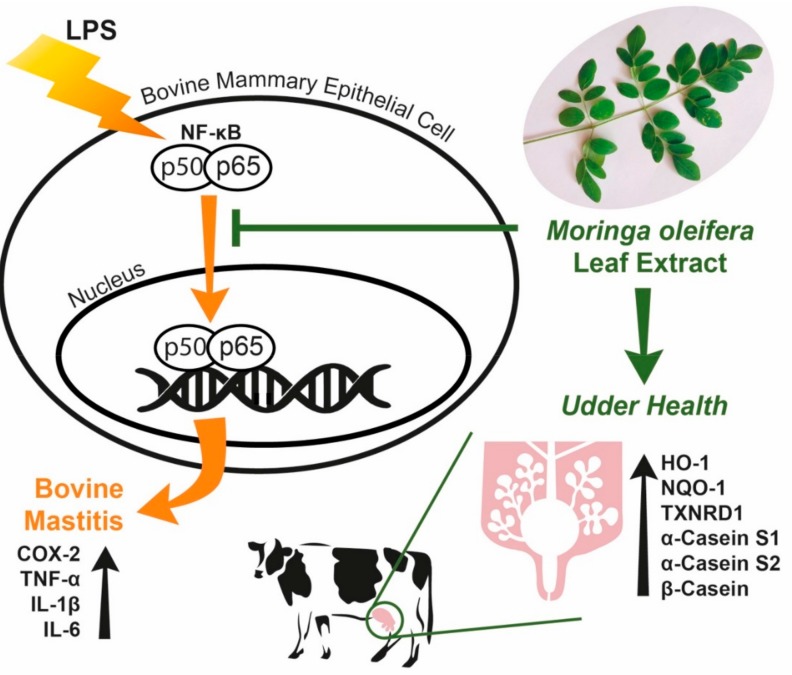
Protective role of moringa extract in LPS-challenged bovine mammary epithelial cells. Moringa extract decreases cellular inflammatory responses induced by LPS in bovine mammary epithelial cells through down-regulation of NF-κB, COX-2, TNF-α, IL-1β and IL-6. Moringa extract increases gene expression of casein and antioxidant proteins.

Our study demonstrated that ME exerted anti-inflammatory effects in bovine mammary epithelial cells by attenuating expression of COX-2 and deactivating NF-κB downregulation of pro-inflammatory cytokines (Figure 8). ME ameliorated cellular oxidative stress by scavenging free radicals, decreasing cellular ROS production, and up-regulating antioxidant genes (Figure 8). More importantly, ME induced mRNA expression levels of milk components such as casein isoforms (Figure 8). Our data suggest that supplementation of moringa in the feed of dairy cows may have beneficial effects in protecting and preventing udder inflammation and improves milk protein production. Further studies using dairy cows are warranted to evaluate moringa as a natural feed additive. 

## Figures and Tables

**Table 1:** Primers used for RT-PCR.

Gene	Sequence 5’-3’
GAPDH	(F) ATG ATT CCA CCC ACG GCA AGT T (R) ACC ACA TAC TCA GCA CCA GCA T
TNF-α	(F) ACG GGC TTT ACC TCA TCT ACT CAC (R) TTG ACC TTG GTC TGG TAG GAG ACT
IL-1β	(F) CCG TAC CTG AAC CCA TCA ACG AAA (R) GGT GTT GGA TGC AGC TCT TCA TCT
IL-6	(F) AGC GCA TGG TCG ACA AAA TCT C (R) AAC CCA GAT TGG AAG CAT CCG T
HO-1	(F) AGG ATT TGT CAG AGG CCC TGA A (R) CAA AGA CGC CAT CAC CAG CTT A
NQO-1	(F) GGT GCT CAT AGG GGA GTT CG (R) GGG AGT GTG CCC AAT GCT AT
TXNRD1	(F) CGG TAT TGC TGG CAA TAG GAA GAG (R) GGC ATA GAT GTA AGG CAC GTT GGT
α-casein S1	(F) GGG AAT CCAT GCC CAA CAG AAA GA (R) GGA ACG TAA TAC CAG GCA CCA GAT
α-casein S2	(F) GGA CGA TAA GCA CTA CCA GAA AGC (R) AGA GTG GGA GTA ATG GGA ACA GCA
β-casein	(F) CCT AAC AGC CTC CCA CAA AA (R) AGA CTG GAG CAG AGG CAG AG
